# Infection, Choice Behavior, and Cross-Infectivity of the Sculpted Damsel Bug, *Nabis roseipennis*, Offered the Tarnished Plant Bug, *Lygus lineolaris*, Infected with Entomopathogenic Nematodes

**DOI:** 10.3390/insects16050475

**Published:** 2025-04-30

**Authors:** James P. Glover, Nathan Spaulding, Marissa I. Nufer, Justin George, Maribel Portilla, Gadi V. P. Reddy

**Affiliations:** 1 USDA-ARS Southern Insect Management Research Unit, 141 Experiment Station Road, P.O. Box 346, Stoneville, MS 38776, USA; nathan.spaulding@usda.gov (N.S.); justin.george@usda.gov (J.G.); maribel.portilla@usda.gov (M.P.); gadi.reddy@usda.gov (G.V.P.R.); 2 Department of Entomology and Plant Pathology, University of Tennessee System, Knoxville, TN 37996, USA; mnufer@vols.utk.edu

**Keywords:** tri-trophic interactions, predation, entomopathogenic nematodes, beneficial insects, crop pests

## Abstract

The tarnished plant bug, *Lygus lineolaris* Palisot de Beauvois (Hemiptera: Miridae), is an economically important pest of row crops worldwide. Ten isolates of entomopathogenic nematodes (EPNs) (Rhabditida: Steinernematidae and Heterorhabditidae) were evaluated against the third instar nymphal stage of the tarnished plant bug and its generalist predator, the sculpted damsel bug, *Nabis roseipennis* Reuter (Hemiptera: Nabidae), one of the most abundant and commonly encountered damsel bugs in cotton and soybean agroecoscapes across the Southeastern United States. The objectives of these experiments were to assess the infectivity of entomopathogenic nematodes (EPN) by direct topical exposure against the sculpted damsel bug and tarnished plant bug, whether the predator prey choice is affected by EPN infection, and if feeding on EPN-infected tarnished plant bug (TPB) prey items could result in cross-infection of the predator. Mortality rates at a concentration of 200 infective juveniles (IJs)/mL significantly differed among isolates and insect species, ranging from 30% to 93% for tarnished plant bugs and from 6% to 38% for sculpted damsels, respectively. The third instars of *L. lineolaris* were more susceptible to the ten nematode isolates than *N. roseipennis*. Higher pathogenicity on the tarnished plant bug and a low mortality potential make strains HbHP88, HbVS, Sc17c+e, and SfSN the most promising candidates for the biological control of *L. lineolaris* under lab and greenhouse conditions while preserving beneficial predators of the Southeastern United States.

## Simple Summary

The tarnished plant bug is a major pest of row crops in the southern United States, causing significant economic losses in cotton and other commodities. This study evaluated the ability of entomopathogenic nematodes to control this pest while assessing the safety of these nematodes for a beneficial insect predator commonly found in crop fields. The objectives were to determine whether nematodes could infect and kill the tarnished plant bug, whether infection alters predator feeding behavior, and whether predators could become infected after consuming infected prey. Laboratory and greenhouse experiments showed that several nematode strains caused high mortality in the pest but had minimal effects on the beneficial predator. Predators consistently avoided infected prey, and even when feeding did occur, no transmission of infection to the predator was observed. These findings demonstrate that selected nematode strains are effective against a key agricultural pest while posing little risk to natural enemies. This research supports integrating nematode-based biological control into crop protection programs and highlights their potential to reduce reliance on chemical insecticides, benefiting both environmental health and sustainable agriculture.

## 1. Introduction

The tarnished plant bug, *Lygus lineolaris* (Palisot de Beauvois), is a major pest of row crops in the southern United States [1]. Highly polyphagous, it feeds on a wide range of host plants, including the most economically important crops grown in the Midsouth [1,2]. The tarnished plant bug has been recognized as a major economic pest of cotton, causing significant fruit loss and economic damage in cotton-producing regions across the mid-South, the Mississippi Delta region, and other areas of the world [3,4,5]. Its pest status is exacerbated by a multivoltine life cycle.

The sculpted damsel bug, *Nabis roseipennis* Reuter (Hemiptera: Nabidae), is among the most abundant and most common of the damsel bugs encountered in cotton and soybean across the Southeastern United States [6,7,8,9]. A complex of *Nabids* spp. are commonly found in traditional row crop agroecosystems, including cotton, soybean, and sorghum, and are predators of many Lepidopteran eggs, aphids, and other small soft-bodied insects [10,11]. Nymphal stages of many *Nabid* spp. can typically consume one or more eggs and/or aphids per day, while more mature instars and adults may consume as many as two dozen prey items [12,13,14,15]. Many species of Nabids, irrespective of the nymphal growth stage, have been reported to feed on all life stages of *Lygus* spp. [16,17].

EPNs in the genera *Steinernema* and *Heterorhabditis* have shown great potential for biological pest control in specific agricultural settings. These nematodes are capable of infecting and killing insect pests, making them an attractive alternative to chemical pesticides, which can have negative effects on the environment and human health [18]. The use of nematodes as biological control agents has been widely researched, and EPNs have emerged as promising candidates [19]. EPNs have been found to be highly effective in controlling a variety of insect pests, including some of the most destructive pests in agriculture, such as the corn rootworm *Diabrotica* spp. and black vine weevil *Otiorhynchus sulcatus* [20,21]. Many species of EPN’s have been shown to be effective against a wide range of insect pests, including white grubs, root weevils, and cutworms [22]. The use of EPNs has been shown to reduce insect pest populations and increase crop yields in specific agricultural settings [23]. Recently, the EPN *S. carpocapsae* was found to be an effective biological control agent against three common mirid pest species within a greenhouse environment [24]. While entomopathogenic nematodes have demonstrated substantial efficacy as soil-dwelling biological control pests, interest has also grown in evaluating predatory hemipterans for their capacity to suppress above-ground pests such as mirids. Furthermore, the Potential of *Nabis americoferus* as a biological control agent of *Lygus lineolaris* in strawberry fields has recently been reported [25].

Furthermore, EPNs have several advantages over traditional chemical insecticides. Nematodes are highly host-specific to their target pests, having considerably less off-target or non-target effects on other organisms, leaving beneficial insects unharmed [26]. They also have a short persistence in the environment, as they are vulnerable to desiccation and predation, which reduces the risk of accumulation in the food chain [27]. In addition, they do not pose a risk of developing resistance in pest populations [28]. Nematodes are less likely to develop resistance to chemical insecticides, which can become less effective over time as pests evolve resistance. Furthermore, nematodes are environmentally friendly and do not leave harmful residues in the soil or water.

The objectives of these experiments were to assess the infectivity (confirmed by nematode emergence) and efficacy (measured as host mortality) of ten strains from two groups of entomopathogenic nematodes (EPNs), *Steinernema* and *Heterorhabditis*, by direct topical exposure against the tarnished plant bug and the generalist cotton predator, the sculpted damsel bug. Here, infectivity is defined as the ability of EPNs to successfully enter and establish within the host, confirmed by nematode recovery from cadavers. Efficacy refers to nematode-induced mortality observed during the post-exposure period, regardless of whether internal infection was visually confirmed. This distinction allowed us to separate the biological success of nematode establishment from the overall pest suppression outcome. Additional objectives were to determine whether the EPN infection status of the prey item influences predator host preference, assessed through predator–prey choice behavior in paired-choice assays, and to investigate whether feeding on EPN-infected prey results in cross-infection of the predator. The goal of this research is to evaluate both the direct impact of EPNs on pest and predator mortality and the behavioral and ecological interactions that may influence their integration into biological control strategies. Greenhouse experiments were conducted to assess cross-infectivity and predation dynamics under semi-field conditions to gain further insight into their practical application.

## 2. Materials and Methods

### 2.1. Insect Source and Rearing

All studies were conducted at the USDA-ARS Southern Insect Management Research Unit, Stoneville, MS. Laboratory-reared TPB and nabid colonies were established from field collections of mixed-age late instar nymphs and adults collected from primarily large stands of seedling Johnsongrass (*Sorghum halepense*) and supplemented with collections from other broadleaf weeds common across the mid-south, including Palmer amaranth (*Amaranthus palmeri*). TPB colonies were reared on a semi-solid autoclavable diet routinely used for the mass rearing of *Lygus lineolaris* [29]. Nabids were reared individually to adulthood in an environmental chamber (Percival Scientific Inc., Perry, IA, USA) at a temperature of 27 ± 0.5 °C, 60 ± 5% RH, and a 14:10 (L:D) h photoperiod. Individuals were supplied with a 42 mL plastic cup containing a small piece of cotton saturated with a 2% sucrose solution as a water source and freshly hatched *Helicoverpa zea* (Boddie) neonates as a live food source.

### 2.2. EPN Cultivation and Insect Inoculation

The ten EPN isolates were multiplied and reared in the last instar larvae of the greater wax moth, *Galleria mellonella* L. (Lepidoptera: Pyralidae), which were obtained from Josh’s Frogs (Owosso, MI, USA) (Table 1) [30]. After the wax moths died from EPN infection, the cadavers were moved to White traps that were also kept at room temperature and covered with a black plastic bag [31,32]. The traps were checked daily for the emergence of IJs, which were collected and stored in tissue culture flasks in a 13 °C refrigerator.

The concentration of infective juveniles was counted using a phase hemacytometer (Hausser Scientific #1475, Waltham, MA, USA), viewed under a Leica S9 stereo microscope (Leica Microsystems, Wetzlar, Germany) and adjusted by adding deionized water to attain a final concentration of 200 IJs per/mL. An aqueous nematode suspension (350 µL) containing 200 IJs per 1 mL was pipetted directly onto the pronotum and mesothoracic regions of individual adult sculpted damsel bugs and one-day-old third instar tarnished plant bug nymphs, which were held separately in 42 mL diet assay cups. The individuals were monitored under a dissecting microscope by removing the plastic lid from the 42 mL assay cup and examined for externally visible signs of EPN infection. Infections were identified based on characteristic changes in body coloration associated with nematode genera: individuals infected with *Heterorhabdtis* spp. typically exhibited a reddish hue due to the proliferation of their symbiotic bacteria *Photorhabdus* spp., whereas individuals infected with *Steinernema* spp. often display a brownish or tan discoloration, resulting from the colonization of *Xenorhabdus* spp. bacteria. These distinct color changes served as a non-invasive visual indicator of successful nematode infection and were used to aid in confirming infectivity prior to cadaver transfer for incubation. After the duration of the experiment (10 d), a representative subset of experimental insects and resulting cadavers were retained and transferred to white traps for incubation to verify nematode infection via the emergence of infective juveniles.

### 2.3. Lab and Greenhouse Prey Preference Trials

Predator behavioral responses to EPN-infected versus uninfected TPB nymphs were evaluated to measure host preference. In addition, we sought to assess the potential of cross-infection, defined as successful EPN transmission to the sculpted damsel bug (SDB) through the consumption of infected prey. Choice trials were conducted using paired-prey assays in laboratory arenas, while cross-infection risk was assessed through post-exposure observation and cadaver recovery in both lab and greenhouse experiments. Predator preference assays were designed according to Avery et al. [33]; in brief, the prey preference behavior of the SDB adults was studied using choice arenas made from sterile Petri dishes (150 × 15 mm: Fisher Scientific, Inc., Waltham, MA, USA) lined with a round VWR^®^ Grade 410 qualitative filter paper (VWR-Avantor, Radnor, PA, USA) and trimmed to fit within the circumference of the bottom petri dish securely, leaving no gaps. Arenas were designed with a central release zone centered on the drawn line using the cap from a 50 mL falcon plastic centrifuge tube placed in the center of each arena.

Third instar tarnished plant bugs were topically treated with aqueous nematode suspensions (350 µL) containing 200 IJs per 1 mL of deionized water. The suspensions were pipetted directly onto the pronotum and mesothoracic regions of insects and held on green bean sections (15 mm) previously triple-washed with 10% NaClO in petri dishes (52 × 5 mm) lined with a round filter paper of similar size for 24 h before being offered to SDB adults. One live 24 h infected tarnished plant bug and one live uninfected nymph (water control) were placed opposite and perpendicular to the principal axis line. Tarnished plant bug nymphs were brushed with a natural hair paint brush (#0) and placed at equal distances from the drawn line (12.7 mm). Featherweight forceps were used to transfer SDB adults from rearing cups to under the 50 mL falcon tube cap with a circular hole punched (20 mm) in the center. Plastic infesting caps were removed once the SDB adult exited the hole in the cap and was freely moving in the arena on the filter paper. Petri dish choice assays were performed in a walk-in environmental chamber with a 16:8 h (L:D) photoperiod, 28 °C, and 15% RH, with arenas placed directly under overhead luminescent light. Predator preference trials resulted in one of three possible outcomes: feeding on an infected TPB prey, feeding on an uninfected TPB nymph, or no choice. A “predation event” was defined as a prolonged stylet insertion and feeding event lasting ≥ 60 s. Preference trials commenced and lasted 20 min once the SDB exited from the hole in the cap and made contact with the filter paper. Trial replicates received freshly cleaned and air-dried Petri dishes and caps (75% alcohol) supplied with a new filter paper. The orientation of the infected and uninfected prey types was randomly assigned to exclude any directional bias.

**Figure 1:**
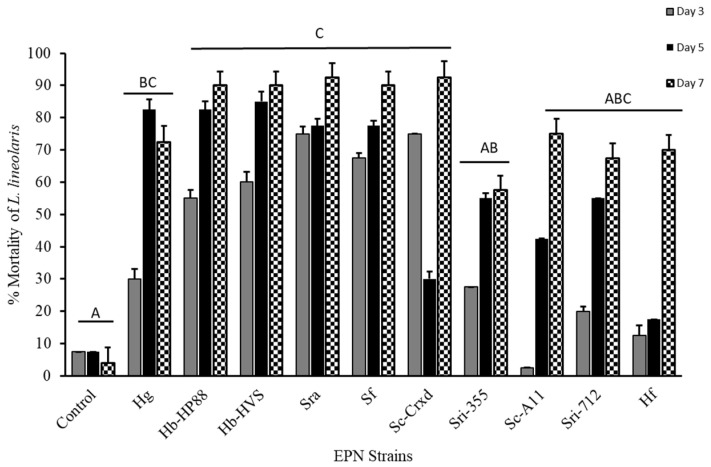
Mortality (mean ± SE) of *L. lineolaris* topically treated with a water control and ten EPN strains: Hg, Hb-HP88, Hb-HVS, Sra, Sf, Sc-Cxrd, Sri-355, Sc-A11, Sri-712, and Hf. Bars with different letters indicate significant differences (*p* < 0.05) between treatments by model contrast analysis based on a generalized linear mixed effect model.

Greenhouse experiments were performed on individual non-Bt cotton plants (DLP1892, Delta Pine, Scott, MS, USA) in two-gallon-sized black nursery greenhouse pots in standard potting media. SDB were released onto greenhouse-grown cotton in the second week of blooming (approx. 12 nodes). Cotton terminals were isolated with insect enclosure bags constructed from organza (22 × 22 × 8 mm~240 µm mesh, JoAnn’s Fabrics, Hudson, OH, USA) that enclosed the plant terminals affixed with pipe cleaners (top five nodes) containing many squaring sites. The second week of bloom (≈55 d) was chosen to reflect not only a physiologically vulnerable growth stage of cotton but also a location that is commonly associated with economic damage. We conducted 20 replicates for the strains with mortality > 70% (strains Hb-HVS, Sra, Sf, Sc-Cxrd) (Figure 1). Plants were infested by carefully opening the enclosure and paint brushing 24 h infected TPB third instar nymphs, one per cage, onto a fully mature and opened terminal leaf and giving them 10 min to acclimate and explore the canopy. Featherweight forceps were used to transfer SDB adults from rearing cups to caged plant terminals, placed on an opposing mature leaf, and the cage was securely fastened after the TPB acclimation period. The cages were vigorously inspected to ensure the insects were not immobilized by the folds in the fabric and were completely confined to the desired nodes of growth. The cages were inspected every 24 h for predated TPB, as evidenced by a shriveled and imploded body indicative of feeding similar to individuals fed on during the petri dish assays. Based on observational records, active feeding or prey handling of TPB nymphs by SDB was noted in approximately 85% (17/20) of the cages during the greenhouse trials.

### 2.4. Data Analysis

All experimental measurements of mortality and infection were analyzed as a randomized complete block with a factorial arrangement in PROC GLM [34] (10 EPN strains examined × third instar tarnished plant bug and adult sculpted damsel bugs exposed plus a water control). Each treatment combination was repeated three times. A Tukey’s mean separation test (α = 0.05) was used to compare results. Individuals who made no prey choice or who never excited the cap were excluded from the analysis. Predator preferences and time data (min) between infected and uninfected prey were analyzed using an *X*^2^ test of independence to estimate the difference in probability of choosing infected ones versus control ones for each strain at each time point, allowing time to be treated as a continuous variable with a non-linear time trend.

## 3. Results

### 3.1. Infection and Mortality of Tarnished Plant Bug Nymphs

The third instar tarnished plant bug mortality at seven days was significantly affected by topical exposure with the ten EPN strains (F = 93.69; df = 10, 418; *p* ≤ 0.0001), with mortality ranging from 57% to 93%. The EPN strains with the highest mortality observed across the study were Sra and Sc-Cxrd, with 94%, 92%, respectively (Figure 1). Although variation in mortality trends was observed across time for different EPN strains (Figure 1), the interaction between strain and time was not statistically significant under our model (*p* > 0.05). A Tukey’s post hoc means separations test (*p* < 0.05) (α = 0.05) determined that strains Hb-HP88, Hb-HVS, SRA, SF, and Sc-Cxrd caused significant mortality at five and seven days post-topical exposure and significant mortality on day three for Sri-355 when compared to strains Sc-A11, Sc-A11, HF, and controls (Figure 1).

### 3.2. Infection and Mortality of Adult Nabids

**Figure 2:**
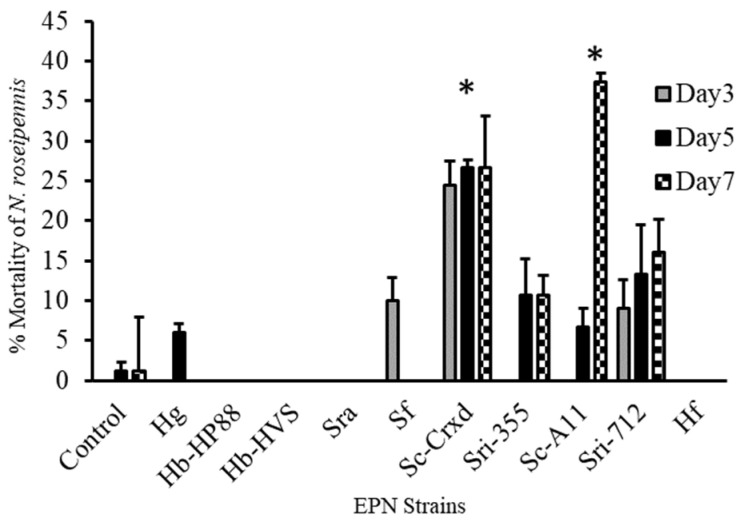
Mortality (mean ± SE) of *N. roseipennis* topically treated with a water control and ten EPN strains: Hg, Hb-HP88, Hb-HVS, Sra, Sf, Sc-Cxrd, Sri-355, Sc-A11, Sri-712, and Hf. Bars with * indicate significant differences (*p* < 0.05) between treatments by model contrast analysis based on a generalized linear mixed effect model.

Adult nabid mortality one-week post exposure to the ten EPN strains was significantly higher for strains Sc-Cxrd and Sc-A11 treatments, and the highest mortality observed ranged from 27 to 38%, respectively (F = 7.19; df = 10, 316; *p* ≤ 0.0001) (Figure 2). A Tukey’s post hoc means separations test (*p* < 0.05) (α = 0.05) determined that nabids exposed to strain Sc-Cxrd experienced significant mortality at all three sampling points and on day seven for strain Sc-A11 post-exposure (Figure 2). All strains caused mortality > 5%, excluding strains Hb-HP88, Hb-HVS, SRA, and HF, where no activity was detected.

### 3.3. Predator Preference

Predator preference for EPN-infected versus uninfected prey was assessed across four nematode strains that demonstrated ≥70% efficacy in prior mortality trials: Hb-HVS, Sra, Sf, and Sc-Cxrd. Each strain was tested in 20 replicates using paired-prey arena assays. Predator behavior was classified into one of three outcomes: feeding on EPN-infected TPB nymphs, feeding on uninfected (control) nymphs, or making no prey choice within the 20 min trial period.

**Figure 3:**
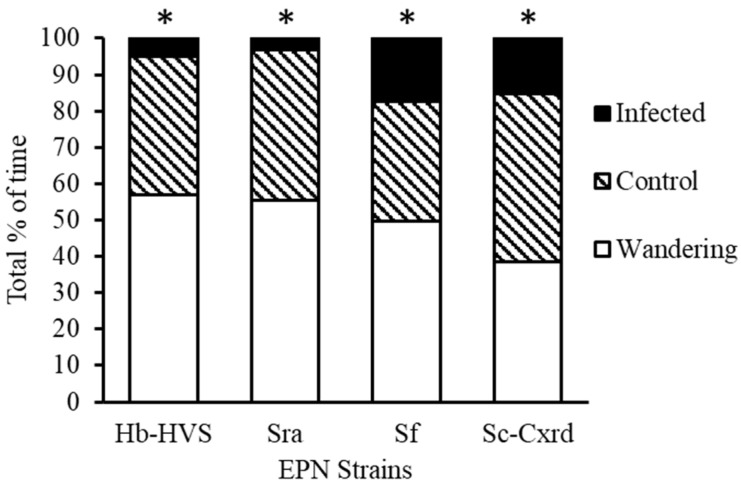
Predator preference of *N. roseipennis* when offered EPN infected versus uninfected third instar *L. lineolaris*nymphs across four EPN strains: Hb-HVS, Sra, Sf, and Sc-Cxrd. * indicates significant differences (*p* < 0.05) between treatments.

In the arena choice assays where nabids had a choice of uninfected tarnished plant bug prey item versus those infected with strain Hb-HVS, 59.4% of SDB made no choice (n = 69), while 32.8% of nabids fed on the un-infected control tarnished plant bug (topical water exposure) (n = 38) and 7.8% (n = 9) fed on an infected prey item (Figure 3). Chi-square tests of independence were used to evaluate differences in prey selection frequencies. For strain Hb-HVS, 59.4% of SDB individuals made no choice (n = 69), 32.8% fed on uninfected prey (n = 38), and 7.8% fed on infected prey (n = 9). Predators significantly avoided infected prey (χ^2^(1, N = 116) = 11.84, *p* < 0.0006), and feeding durations were shorter on infected prey (mean = 45 ± 13 s) than on uninfected prey (mean = 321 ± 17 s; range 60–900 s).

When offered prey infected with strain Sra, 56% of predators made no choice (n = 105), 40.6% fed on uninfected prey (n = 76), and 3.2% fed on infected prey (n = 6) (Figure 3). Infected prey were again significantly avoided (χ^2^(1, N = 187) = 13.55, *p* < 0.0002), with feeding durations averaging 30 s versus 452 ± 25 s on uninfected prey.

For strain Sf, 49% of predators made no choice (n = 92), 33% fed on uninfected prey (n = 62), and 17% fed on infected prey (n = 33) (Figure 3). Despite increased feeding on infected prey compared to other strains, avoidance remained significant (χ^2^(1, N = 187) = 14.72, *p* < 0.0001). Feeding durations on infected prey averaged 153 s versus 565 ± 15 s on controls.

With strain Sc-Cxrd, 39% of predators made no choice (n = 73), 44% fed on uninfected prey (n = 82), and 17% fed on infected prey (n = 32) (Figure 3), also showing significant avoidance (χ^2^(1, N = 187) = 11.63, *p* < 0.0006). The feeding duration averaged 62 s on infected prey compared to 528 ± 37 s on uninfected prey. The longest feeding durations on infected prey were observed with strains Sf and Sc-Cxrd (204 s and 180 s, respectively).

### 3.4. Greenhouse No Choice Assay and Cross-Infectivity

**Figure 4:**
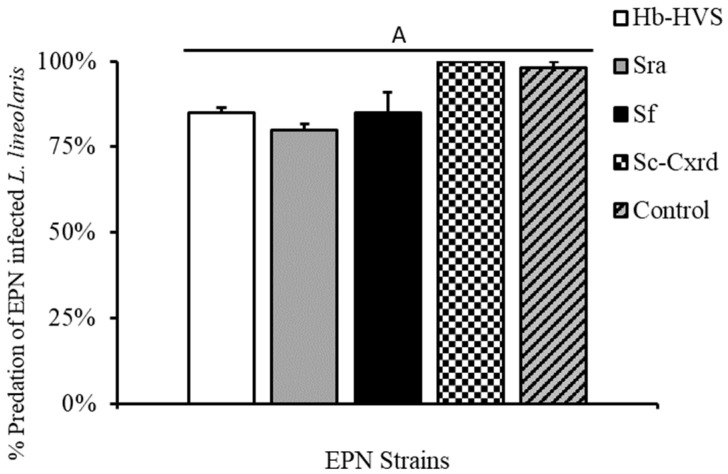
Percent predation by *N. roseipennis* (mean ± SE) of *L. lineolaris* infected with four EPN strains: Hb-HVS, Sra, Sf, and Sc-Cxrd, as well as a water control. Bars with different letters indicate significant differences (*p* < 0.05).

Nabids individually infested on greenhouse-grown cotton plants consumed >80% of infected prey items in caged no-choice assays across the four strains tested (Figure 4). To evaluate cross-infection potential, SDB individuals that fed on infected TPB prey (n = 81) were retained post-experiment and incubated in White traps. No nematode emergence was observed from any predator cadavers across all four strains, indicating no evidence of cross-infection under experimental conditions (Table 1).

## 4. Discussion

The present study provides novel insights into the interactions among entomopathogenic nematodes (EPNs), a key pest (*Lygus lineolaris*) and an important generalist predator (*Nabis roseipennis*). Historically recognized for their environmental safety and specificity towards target pests, EPNs represent promising biological control agents [35]. Our findings expand the understanding of their ecological role by exploring direct infectivity to both pests and predators, predator choice behavior influenced by prey infection, and the risk of cross-infectivity.

**Figure 5:**
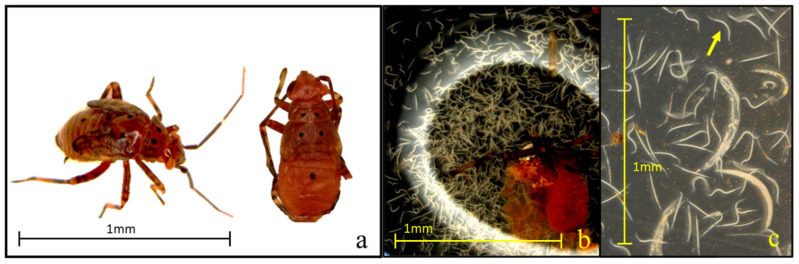
(**a**–**c**) Visual symptoms of *Lygus lineolaris* infection by entomopathogenic nematodes (EPNs). Third instar *L. lineolaris* nymph displaying a characteristic brownish discoloration indicative of an infection by *Steinernema* spp. (**a**), dissected abdomen of an infected nymph showing nematode proliferation within the body cavity (**b**), view of developing juvenile (arrow) and adult nematodes recovered from infected cadaver tissue (**c**).

All ten tested EPN strains demonstrated significant pathogenicity against third instar tarnished plant bugs, achieving mortality rates above 60% within seven days. Notably, the isolates Hb-HP88, Hb-HVS, Sra, Sf, and Sc-Cxrd caused substantial mortality (>50%) by day three, demonstrating their rapid efficacy. Steenman et al. [24] observed similar susceptibilities in mirid pests to specific *Steinernema* strains. Cadavers were assessed for EPN infection using body coloration, a practical and widely accepted diagnostic approach in EPN studies, where reddish or brownish hues are indicative of infection by *Heterorhabditis* or *Steinernema*, respectively (Figure 5). Although no molecular confirmation was performed, strict strain-specific handling protocols were followed to reduce the risk of cross-contamination.

Adult sculpted damsel bugs displayed considerably lower susceptibility, with mortality significantly elevated only by strains Sc-Cxrd and Sc-A11 (27–38%). Importantly, no significant mortality was induced by isolates Hb-HP88, Hb-HVS, Sra, and Hf. This selective susceptibility demonstrates the potential for targeted pest management while conserving beneficial predators [35]. Consequently, isolates Hb-HVS, Sra, Sf, and Sc-Cxrd were identified as promising candidates, balancing high pest mortality with minimal non-target impacts.

Predator choice assays revealed a clear behavioral avoidance of EPN-infected prey by *N. roseipennis*. When presented with healthy versus infected prey, damsel bugs significantly preferred uninfected tarnished plant bugs and drastically reduced their feeding duration upon contact with infected individuals. Cadavers hosting the nematode/bacteria complex have been shown to be highly deterrent for detritivorous insects and were first demonstrated with the EPN *Photorhabdus luminescens* (Heterorhabditidae) and workers of *Linepithema humile* (Hymenoptera: Formicidae) [36]. Mertz et al. [37] demonstrated that the predator *Calosoma granulatum* actively avoided feeding on infected larvae of *Spodoptera frugiperda* when given a choice of healthy versus infected prey types. The avoidance response observed in laboratory arenas suggests a possible chemical or behavioral cue that alerts predators to infected prey, which warrants further investigation under field conditions. Despite the observed behavioral avoidance, no damsel bug exhibited signs of infection across all tested nematode isolates, irrespective of the feeding duration on infected prey. This finding suggests minimal risk of cross-infection, enhancing the ecological compatibility of EPN applications with predator-based biological control programs [12]. Greenhouse no-choice experiments further confirmed predator safety; damsel bugs effectively predated EPN-infected tarnished plant bugs (>80% consumption) without subsequent nematode transmission.

This study demonstrates that selective EPN isolates (Hb-HVS, Sra, Sf, Sc-Cxrd) possess significant potential for integration into IPM strategies targeting tarnished plant bugs. Their usage can effectively reduce pest populations while safeguarding beneficial predators like *N. roseipennis*. Future field research should focus on identifying the chemical cues associated with infected prey avoidance and optimizing EPN application methods through encapsulation and UV protection technologies to maximize efficacy under environmental conditions.

## Figures and Tables

**Table 1:** List/codex of entomopathogenic species of nematodes (EPNs) used in a series of experiments presented.

EPN Species	EPN Origin	EPN Strain	Codex
*Heterorhabditis georgiana*	Georgia, USA	Kesha	Hg
*Heterorhabditis bacteriophora*	Utah, USA	HP88	Hb-HP88
*Heterorhabditis bacteriophora*	Georgia, USA	HVS	Hb-HVS
*Steinernema rarum*	Combined from isolates found in Louisiana, USA and Mississippi, USA	17c+e	Sra
*Steinernema feltiae*	France	SN	Sf
*Steinernema carpocapsae*	Arkansas, USA	Cxrd	Sc-Cxrd
*Steinernema riobrave*	Texas, USA	355	Sri-355
*Steinernema carpocapsae*	Georgia, USA	A11	Sc-A11
*Steinernema riobrave*	Texas, USA	7–12	Sri-712
*Heterorhabditis floridensis*	Georgia, USA	K22	Hf

## Data Availability

The data presented in this study are included in the article. Further inquiries can be directed to the corresponding author.
